# Expert Opinion About the Pharmacoeconomic Edge of Low-Cost Dapagliflozin in Type 2 Diabetes Mellitus in Indian Clinical Settings

**DOI:** 10.7759/cureus.19194

**Published:** 2021-11-01

**Authors:** Kamal Sharma, A B Chandorkar, Rajiv Kovil, S Venkataraman, KAV Subrahmanyam, Parthasarathi Mandal, Jasjeet Wasir, Mahesh Abhyankar, Ashish Prasad, Prashant S Sarda

**Affiliations:** 1 Department of Cardiology, Dr. Kamal Sharma Cardiology Clinic, Ahmedabad, IND; 2 Department of Cardiology, Ruby Hall Clinic, Pune, IND; 3 Department of Diabetology and Endocrinology, Nanavati Super Specialty Hospital, Mumbai, IND; 4 Department of Diabetology, Soorya Hospital, Chennai, IND; 5 Department of Endocrinology, King George Hospital, Visakhapatnam, IND; 6 Department of Diabetes and Endocrinology, AMRI Hospitals, Kolkata, IND; 7 Department of Endocrinology, Medanta Hospital, Gurgaon, IND; 8 Scientific Services, USV Private Limited, Mumbai, IND

**Keywords:** dapagliflozin, cost effectiveness, cost-utility analysis, t2dm, expert opinion

## Abstract

Background

This study aimed to understand the attitudes, beliefs, and concerns of physicians across India regarding the economic burden of diabetes and subsequently the cost-effectiveness of low-cost dapagliflozin for the management of patients with uncontrolled type 2 diabetes mellitus (T2DM) on background metformin therapy in Indian clinical settings.

Method

A cross-sectional questionnaire survey was conducted among physicians treating people with T2DM with or without complications. The questions covered the general aspects of affordability and adherence to diabetes medications as well as specific details of low-cost dapagliflozin and its cost-effectiveness.

Results

In total, 844 physicians provided a response to the survey questionnaire. The physicians who participated in the study included diabetologists, endocrinologists, cardiologists, consulting physicians, and family physicians. A majority of the physicians (53%) opined that only 10%-30% of their patients can afford the cost of newer antidiabetic medicines, while 25% of the physicians mentioned that <10% of their patients had issues related to affordability. Further, 39% of the physicians opined that 20%-40% of their patients discontinue the medicines due to high cost. Most of the physicians (95%) agreed that due to the low cost of dapagliflozin, it can be used for the primary prevention of heart failure in patients with T2DM in India. Similarly, 98% of the physicians agreed that it can be used for the treatment of heart failure in patients with or without T2DM in India. A majority of these physicians (93%) responded that switching from expensive sodium/glucose cotransporter-2 inhibitors (SGLT2i) to low-cost dapagliflozin is a long-term cost-effective management of T2DM. In total, 98% of the physicians agreed that low-cost dapagliflozin has the characteristics of an ideal SGLT2i because of its metabolic benefits, cardioprotection, nephroprotection, and potential cost-effectiveness.

Conclusion

This survey-based study indicates that dapagliflozin is an effective and cost-saving therapy for patients with T2DM and complications. Low-cost dapagliflozin can revolutionize the treatment of T2DM in the Indian setting.

## Introduction

Diabetes mellitus is a chronic metabolic disorder, one of the major causes of morbidity, mortality and needs lifelong treatment [1]. People living with diabetes are at risk of developing a number of serious and life-threatening complications, leading to an increased need for medical care, reduced quality of life, and undue stress on families. Diabetes and its complications, if not well managed, can lead to frequent hospital admissions and premature death [2].

With this rising prevalence, it can be very well noted that diabetes is an epidemic that does not regard any financial status or any ethnic background. The complications associated with diabetes result in a recurrent encounter with health care systems, thus impacting the financial load of an individual [3]. The cost of treatment of diabetes and its complications tremendously affect both households as well as national expenses. Economic analyses of diabetes care in India found that the cost of providing routine care is only a fraction of the overall cost and is perhaps still manageable. The overall direct and indirect costs escalate with lifelong health problems and economic consequences to the individual, their family, and society, particularly due to the onset of the micro- and macrovascular complications of the disease [4]. For a country like India, where there is a lack of insurance schemes or policies for diabetes treatment, a major proportion of patients with diabetes are impacted by a substantial cost burden from out-of-pocket expenses [5]. The burden of diabetes in India needs to focus on cost-effective measures such as early screening, tight metabolic control, monitoring of risk factors, and assessment of organ damage and economic management solutions [4].

Currently, the goal of the management of type 2 diabetes mellitus (T2DM) is to prevent diabetes-related complications by controlling blood glucose levels [6]. The use of metformin as a first-line therapy is known to manage hyperglycemia, thus decreasing complications of T2DM. However, as the disease progresses, it could be beneficial to add other antidiabetic agents to the ongoing metformin therapy for maintenance of euglycemia. The advent of many novel therapies for the treatment of T2DM increases the choice of drugs used as a second-line treatment. The sodium-glucose co-transporter 2 (SGLT2) inhibitors have been known to act by stimulating glucose excretion in the urine and is the most recent class of therapy that has been approved for T2DM management [7]. Thus, it has been reported that early treatment with a combination of metformin and SGLT2 inhibitors could be effective in rapidly achieving HbA1c targets with weight loss and better blood pressure control. One such SGLT2i with potential benefits in major aspects of diabetes management is dapagliflozin [8]. DECLARE-TIMI 58 trial found that dapagliflozin reduced cardiovascular deaths by 17% and hospitalizations for heart failure by 27% in patients with T2DM with cardiovascular (CV) risk factors or CV disease (CVD) [9]. DAPA-HF trial reported the efficacy of dapagliflozin in patients with heart failure with reduced ejection fraction regardless of a diabetes diagnosis compared to placebo treatment. Hospitalizations for heart failure alone were reduced by 30%. These benefits were comparable in patients with or without diabetes in the study based on a subgroup analysis [10]. Recently, the DAPA-CKD trial demonstrated that among patients with chronic kidney disease (CKD), regardless of the presence or absence of diabetes, the risk of a composite of a sustained decline in the estimated GFR of at least 50%, end-stage kidney disease, or death from renal or cardiovascular causes was significantly lower with dapagliflozin than with placebo [11].

Several studies relating to the cost-effectiveness of dapagliflozin have shown the cost-utility of add-on dapagliflozin treatment in patients with or without diabetes [12,13]. From October 2020, dapagliflozin is available in India as a low-cost generic drug and the resulting drastic reduction in the price is benefiting many eligible patients. Generic drugs are equivalent to the brand formulation if they have the same active substance, the same pharmaceutical form and the same therapeutic indications and similar bioequivalence with respect to the reference medicinal product [14]. In the background of the introduction of low-cost dapagliflozin, pharmacoeconomics plays a significant role to help in decision-making to improve medication adherence. There are various pharmacoeconomic models used for deciding the cost of a drug or therapy [15,16]. This survey-based study aimed to analyze the cost-effectiveness of low-cost dapagliflozin for the management of patients with uncontrolled T2DM on background metformin therapy in Indian clinical settings.

## Materials and methods

This survey was designed to understand the attitudes, beliefs, and concerns of physicians across India regarding the economic burden and subsequently cost-effectiveness of newer antidiabetic agents. The responses from the physicians aimed to yield data on medicine affordability and adherence, cost of complications, and the usefulness of dapagliflozin in managing these complications. Various pharmacoeconomic models used for deciding the cost of a drug or therapy are summarized in Table 1.

**Table 1:** Pharmacoeconomic analysis based on types of costs and outcomes.

Cost-minimization analysis (CMA)	Analysis of the least expensive therapy that causes equivalent health outcomes
Cost-effectiveness analysis (CEA)	Comparison of therapies regarding costs and outcomes, e.g., how much money can bring about reduced mortality or morbidity.
Cost-utility analysis (CUA)	A type of CEA that compares costs with health outcomes with regards to their utility and mortality, which is expressed in quality-adjusted life years (QALYs).
Cost-consequence analysis (CCA)	A type of CEA that evaluates costs and health outcomes in different categories so that there is no overlap between them.
Cost-benefit analysis (CBA)	Compares costs and health benefits (and risks), all of which are quantified in common monetary units.

The respondent physicians across India were requested to go through published original articles and publications at the start of the survey. The respondents were requested to answer the electronic survey based on their clinical experiences with patients having diabetes, while some of the responses were collected during round-table meetings conducted in a group of 10-15 physicians. The questionnaire consisted of 12 questions based on the role of dapagliflozin in primary prevention, affordability and cost-effectiveness in micro- as well as macrovascular complications.

Completed questionnaires from all the participants were compiled for analysis. A descriptive analysis of the collected data was performed by trained personnel and the results were expressed in percentages based on the number of responses obtained for each question. All respondents participated in the survey voluntarily, and no incentives were provided to the physicians for their participation.

## Results

A total of 2000 email invitations were sent to clinicians inviting them to participate in this anonymous survey of which 844 clinicians completed the survey. Of them, 650 (77%), 30 (3.5%), 41 (4.8%), 67 (7.9%), and 56 (6.6%) were consulting physicians, diabetologists, endocrinologists, cardiologists, and family physicians, respectively. Physicians belonged to various zones of India including east zone (119[14.09%]), west zone (232 [27.48%]), south zone (291 [34.47%]), north zone (103 [12.20%]), and central zone (99 [11.72%]). Key findings from the clinician’s responses are summarized in Table 2.

**Table 2:** Key expert opinion from the survey.

Key expert opinion	
Affordability and adherence of newer antidiabetic medications	The newer antidiabetic agents have concerns of affordability in the majority of the patients in T2DM.
	This non-affordability of newer antidiabetic agents leads to discontinuation of medicines.
Cost of diabetes and its complications	The total cost of diabetes without complications is not less than Rs 40,000 (≈ $ 547.5) per annum.
	The cost of diabetes increases with rise in complications.
	Only 10%-30% of T2DM patients with renal impairment can afford the cost of dialysis.
Cost-effectiveness of low-cost dapagliflozin in T2DM and its complications	Low-cost dapagliflozin may have a significant advantage in the primary prevention of heart failure with or without T2DM and in CKD patients in India.
	Low-cost dapagliflozin reduces the overall cost of diabetes management in India.
Overall benefits of low-cost dapagliflozin in T2DM and its complications	Low-cost dapagliflozin has the characteristics of an ideal SGLT2i such as metabolic benefits, cardioprotection and nephroprotection.
	Low-cost dapagliflozin has strong evidence of cardiorenal benefits as compared to remogliflozin.

Affordability and adherence to newer antidiabetic medicines

**Figure 1:**
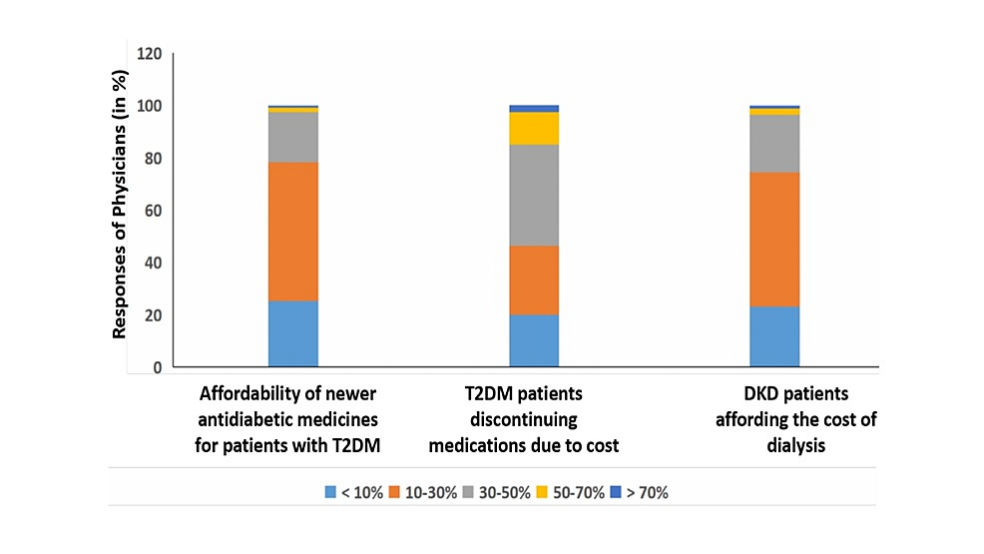
Physicians’ opinions about affordability and adherence of newer antidiabetic medicines. T2DM: type 2 diabetes mellitus; DKD: diabetic kidney disease.

Regarding the affordability of newer antidiabetic medicines, a majority of the physicians (53%) opined that only 10%-30% of their patients can afford the cost, while 25% and 19% of physicians mentioned that <10% and 30%-50% of their patients can afford the cost, respectively (Figure 1).

A total of 39% of physicians opined that 20%-40% of their patients discontinue the medicines due to cost, while 26%, 17%, and 12% of the physicians mentioned that 10%-20%, 5%-10%, and 20%-40% of their patients do not adhere to medicines due to high cost, respectively (Figure 1).

Cost of diabetes and its complications

**Figure 2:**
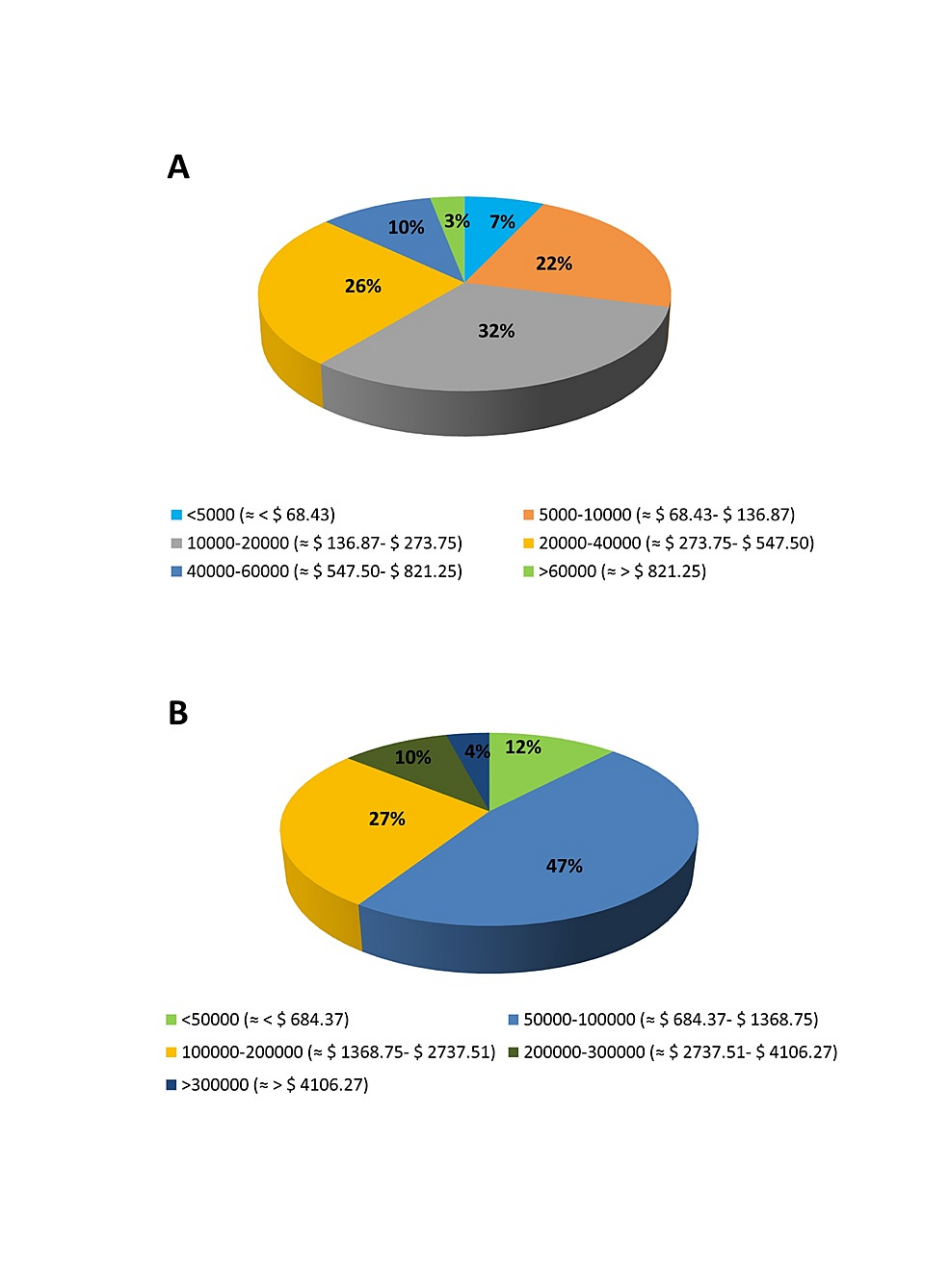
Physicians’ opinions about cost of diabetes and its complications. (A) Distribution of responses from physicians according to the total cost of diabetes care (in INR, including medicines, investigations, consultation fee) in a person with T2DM without complications. (B) Physicians’ responses about average cost (in INR) of single hospitalization for heart failure in patients with T2DM in India. Data shown in brackets are USD conversion rate at 1st January 2021 ($ 1= 73.059 INR) according to https://www.xe.com/currencytables/?from=USD&date=2021-01-01#table-section

The survey results showed that a majority of the physicians (58%) recognize that the economic burden of diabetes without complications ranges from 10,000-40,000 INR (≈ $ 136.87 -$547.50) per annum (Figure 2A). The total cost of single hospitalization due to heart failure in Indian settings is 50,000-1,00,000 INR (≈ $ 684.37-$ 1,368.75) and 1,00,000-2,00,000 INR (≈ $ 1,368.75 -$2,737.51) as opined by 47% and 27% of physicians, respectively. (Figure 2B). Even in the case of T2DM with renal failure, 51%, 23%, and 22% of the respondent physicians agreed that only 10%-30%, <10%, and 30%-50% of their patients can afford the cost of dialysis in T2DM, respectively (Figure 1).

Cost-effectiveness of low-cost dapagliflozin in T2DM and complications

Overall, 95% of physicians agreed that the low cost of dapagliflozin can be beneficial for primary prevention of heart failure in T2DM in India; of which, 70% of physicians strongly agreed to the same. A similar proportion of physicians (98%) also agreed or strongly agreed that low-cost dapagliflozin can be used for the treatment of heart failure with or without T2DM in India.

**Figure 3:**
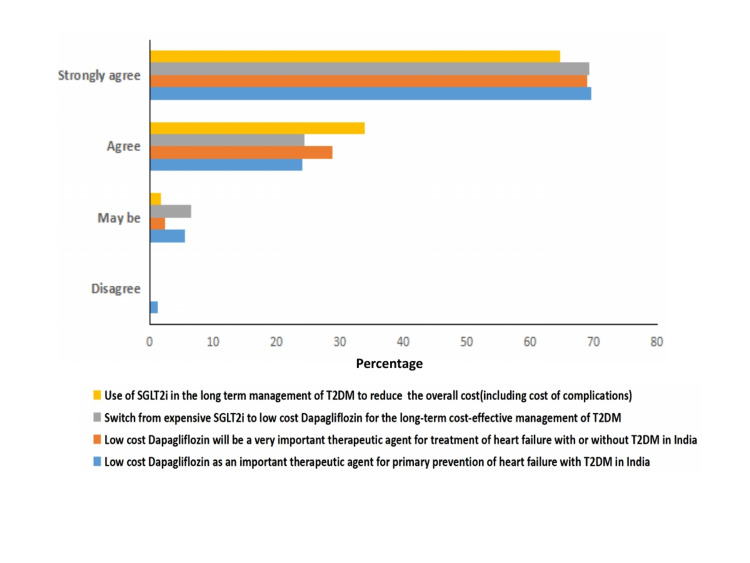
Physicians’ responses about cost-effectiveness of dapagliflozin in T2DM and complications. SGLT2i: sodium/glucose cotransporter-2 inhibitors; T2DM: type 2 diabetes mellitus.

Further, a majority of the physicians (98%) were of the opinion that dapagliflozin can reduce the overall cost (including the cost of complications) in the long-term management of T2DM. A majority of these physicians (93%) also responded that switching from expensive SGLT2i to low-cost dapagliflozin will lead to long-term cost-effective management of T2DM (Figure 3).

Overall benefits of low-cost dapagliflozin in T2DM and complications

**Figure 4:**
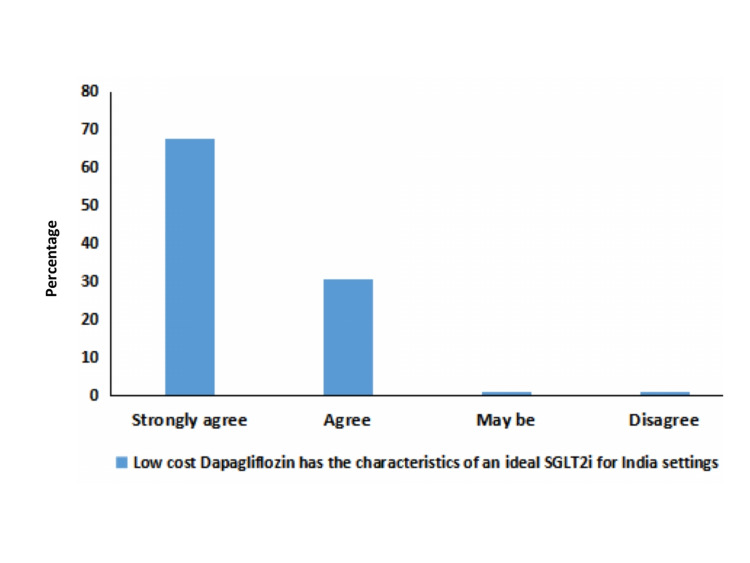
Physicians’ responses about overall benefits of dapagliflozin in T2DM and complications. SGLT2i: sodium/glucose cotransporter-2 inhibitors; T2DM: type 2 diabetes mellitus.

According to this survey, 98% of the physicians agreed that low-cost dapagliflozin has the characteristics of an ideal SGLT2i because of its metabolic benefits, cardioprotection, nephroprotection, and potential cost-effectiveness (Figure 4). When compared to low-cost SGLT2i like remogliflozin, it was observed that 95% of physicians preferred dapagliflozin over remogliflozin due to the protective cardiorenal outcomes of dapagliflozin.

**Figure 5:**
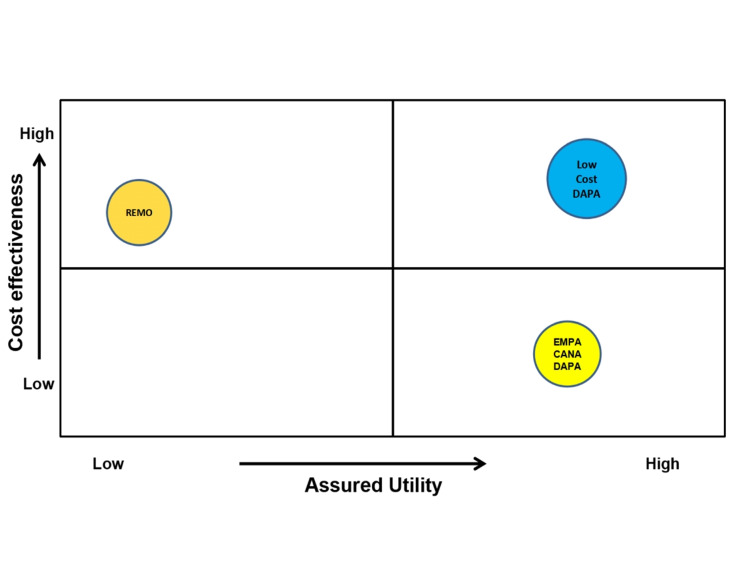
Cost-utility analysis (dapagliflozin vs other SGLT2i). DAPA: dapagliflozin; EMPA: empagliflozin; CANA: canagliflozin; REMO: remogliflozin; SGLT2i: sodium/glucose cotransporter-2 inhibitors.

Based on the assured utility and cost-effectiveness of low-cost dapagliflozin when compared to other high-cost and low-cost SGLT2i, a cost-utility matrix was created that gives a complete picture of where low-cost dapagliflozin stands with respect to cost-effectiveness and potential benefits (Figure 5). This matrix is the outcome of the pharmacoeconomic models that analyzed the health benefits of low-cost dapagliflozin with respect to the overall cost of management of diabetes and its complications.

## Discussion

In the Indian clinical setting, the cost of diabetes matters the most as diabetes is seen in patients across all socio-economic strata, lack of medical insurance and medicines being out-of-pocket expenses for many. This rising cost of treatment for diabetes and its complications includes the cost involved in medicines, investigations, consultation fees, hospitalization etc. Therefore, a significant number of patients cannot afford the cost of new antidiabetic drugs. Further, the affordability of drugs also plays an important role in medication adherence. The branch of pharmacoeconomics plays an important role in determining the costs and benefits of drug therapy. There are several pharmacoeconomic analyses that evaluate health consequences and also the cost of various intervention strategies (Table 1) [15]. These analyses take into consideration the provider, payer, patient, and broader aspects of society. Proper application of pharmacoeconomics will allow the pharmacy practitioners and administrators to make better and more informed decisions regarding the products and services they provide [17].

The main aim of this study was to understand and evaluate the pharmacoeconomics of diabetes treatment with and without complications and how the cost of newer antidiabetic medicines can be effectively compared with the health consequences and quality of life. This survey provides new data on physicians' views of the issue that could be further accepted by patients and health care providers.

First, although the physicians who participated in the survey were from various cities and towns across India, tending to patients of different financial statures, there was consistency in their responses concerning the affordability and adherence of newer anti-diabetic medications. The physicians were of the opinion that a majority of the patients cannot afford the newer therapies, irrespective of their financial background. This makes an impact on the adherence of medications since non-affordability can lead to the discontinuation of medications in a large proportion of patients. Further, the presence of complications like renal impairment and heart failure in patients with T2DM also burns the pockets of many patients. These observations suggest that the physicians are aware of the financial constraints of an individual and their family in context with the treatment of diabetes and its management. Additionally, as per the recent (2020) World Bank data, the GDP of India is 1,900.7 which is much lower as compared to other countries demonstrating non-affording possibilities [18].

As a result, it is of substantial importance to understand the cost-effectiveness of antidiabetic medicine like dapagliflozin, once available in generic form. In the present survey-based study, the physicians had a unanimously positive response to the acceptance of low-cost dapagliflozin. The majority of these physicians, who are the primary health care providers of patients with T2DM, suggested that in their clinical practice, they have experienced the benefits of low-cost dapagliflozin in primary prevention of heart failure in patients with T2DM and even as a very important therapeutic agent for the treatment of heart failure in patients with or without T2DM in India. Evidence indicates that there is a higher proportion of patients with heart failure and T2DM in the Asian population as compared to Caucasians [19]. Therefore, cost concern for new medications is greater in low or low-middle-income countries [20]. Dapagliflozin can be used for the treatment of heart failure with or without T2DM in India, emphasizing that dapagliflozin can also be used in non-diabetic patients for the management of heart failure.

Although the physicians and patients believe that newer treatments might be effective, they are simply too expensive. However, the results of this study confirm that low-cost dapagliflozin is a model of cost-utility analysis and it positively compares the low cost with health outcomes thereby increasing the quality of life. The physicians also had the suggestion of switching to low-cost dapagliflozin as an alternative to high-cost SGLT2i, keeping into consideration the health benefits of reduced mortality and morbidity.

Finally, because of all the benefits of cost and positive health outcomes, such as metabolic benefits, cardioprotection and nephroprotection of dapagliflozin, the physicians were of the opinion that low-cost dapagliflozin can be a suitable and considerably beneficial option for the treatment of diabetes and its complications. Dapagliflozin is a well-studied molecule with strong evidence of cardiorenal benefits and the cost-effectiveness also makes it a drug of choice for many patients with T2DM. The results of this survey are in accordance with previous cost-effectiveness studies of dapagliflozin where it is demonstrated that dapagliflozin is a cost-saving therapy for patients with T2DM [21]. Dapagliflozin lowers the incidences of micro-and macro-vascular complications in people with or without T2DM. Consequently, there is an improvement in the quality of life of T2DM patients when treated with dapagliflozin due to its metabolic aspects, impact on weight (weight reduction), and cardiorenal protection [21].

Limitations

This study was not a real-world study. The physicians who participated in the survey might have ambiguity about their experiences. The attitudes and perceptions of the respondent physicians are subject to change over the course of time. The findings of the survey must not be generalized to a specific section of the society as the respondents were from different societal backgrounds. A prospective study should be initiated to measure the cost-effectiveness and cost utilization of low-cost dapagliflozin in T2DM. 

## Conclusions

This survey-based study suggests that dapagliflozin is an effective and cost-saving therapy for patients with T2DM and associated complications. The valuable opinions from physicians who participated in this study suggest that low-cost dapagliflozin is an effective, safe, and reasonable choice for the treatment of T2DM patients with multiple risk factors and comorbidities such as established CVD (with and without prior heart failure and/or CKD). Therefore, low-cost dapagliflozin can revolutionize the treatment of T2DM in the Indian setting. 
